# The Functional Role of Prion Protein (PrP^C^) on Autophagy

**DOI:** 10.3390/pathogens2030436

**Published:** 2013-06-26

**Authors:** Hae-Young Shin, Jae-Min Oh, Yong-Sun Kim

**Affiliations:** 1 Ilsong Institute of Life Science, Hallym University, 1605-4 Gwanyang-dong, Dongan-gu, Anyang, Gyeonggi-do 431-060, Republic of Korea; E-Mails : shilysea@hallym.ac.kr (H.Y.S.); jmoh76@hallym.ac.kr (J.M.O.); 2 Department of Microbiology, College of Medicine, Hallym University, 1 Okcheon-dong, Chuncheon, Kangwon-do 200-702, Republic of Korea

**Keywords:** prion, *Prnp*-deficient, prion diseases, autophagy, autophagic flux, oxidative stress

## Abstract

Cellular prion protein (PrP^C^) plays an important role in the cellular defense against oxidative stress. However, the exact protective mechanism of PrP^C^ is unclear. Autophagy is essential for survival, differentiation, development, and homeostasis in several organisms. Although the role that autophagy plays in neurodegenerative disease has yet to be established, it is clear that autophagy-induced cell death is observed in neurodegenerative disorders that exhibit protein aggregations. Moreover, autophagy can promote cell survival and cell death under various conditions. In this review, we describe the involvement of autophagy in prion disease and the effects of PrP^C^.

## 1. Introduction

Normal cellular prion protein (PrP^C^) is a glycosylphosphatidylinositol (GPI)-anchored glycoprotein on the extracellular surface that is highly expressed in the central nervous system (CNS), in particular by neurons [1,2,3,4]. Normal PrP^C^ is converted into the abnormal scrapie isoform, PrP^Sc^, when a portion of its α-helical coil structure is refolded into a β-sheet [3,4]. This structural change confers partial resistance to proteolytic degradation and detergent insolubility to PrP^Sc^ [5,6]. Prion diseases, such as scrapie and bovine spongiform encephalopathy (BSE) in animals and Creutzfeldt-Jakob disease (CJD) in humans, are neurodegenerative conditions characterized by the accumulation of this altered PrP isoform, PrP^Sc^ [7,8]. 

A certain degree of neurodegeneration in these diseases is induced by autophagic cell death, which is characterized by the accumulation of autophagic vacuoles including preautophagosomes, autophagosomes, and autophagolysosomes [9,10,11]. During this process, the cytoplasmic form of microtubule-associated light chain 3 (LC3-I, 18 kDa) is converted into the preautophagosomal and autophagosomal membrane-bound form of LC3 (LC3-II, 16 kDa), which is the most reliable marker for the activation of autophagy [12]. For normal cell growth and development, protein synthesis and organelle biogenesis are balanced against protein degradation and organelle turnover [9]. The major pathways for the degradation of cellular constituents are autophagy and cytosolic turnover by the proteasome [9]. Autophagy plays an important role in cellular homeostasis, *i.e*., the turnover of intracellular organelles and long-lived protein; however, excessive autophagy has been proposed to cause cellular destruction [9,10]. Autophagy is observed in all nucleated cell types that have been analyzed, and the process is essentially the same in yeast, plant, and animal cells [13,14,15]. However, the functions of autophagy, particularly in neurons, are still largely unknown. In this review, we focus on the possible role that PrP^C^ play in the autophagy pathway.

## 2. The Functional Role of PrP^C^ on Autophagy *in vitro*

The PrP^C^ is encoded by the *Prnp* gene, which is highly conserved in a wide range of mammalian species. PrP^C^ is highly expressed throughout the CNS but has also been observed in other organs [1,2,16]. The exact physiological functions of PrP^C^ in the CNS are unclear, but several reports demonstrated that this protein is involved in various biological processes. 

Autophagy is a lysosomal degradation process that is associated with the intracellular turnover of cytoplasmic proteins and organelles [9]. Autophagy is involved in cell survival in response to nutrient deprivation and is also associated with various diseases [17,18].

In prion diseases, the appearance of autophagic vacuoles was first observed in neurons in experimental rodent models affected by transmissible spongiform encephalopathies (TSEs) and in scrapie prion-infected cultured cells [19,20,21]. Autophagic vacuoles were identified in neurons in experimentally induced scrapie and human transmissible encephalopathies [22,23]. It was therefore proposed by Liberski that autophagy could contribute to the spongiform degeneration that is a pathological hallmark in brains affected by prion diseases [22]. In neurons from scrapie-infected mice, increased levels of stimulator of chondrogenesis 1/scrapie responsive gene 1 (SCRG1) are detected in autophagic vacuoles [24]. More recently, it was reported that the pharmacological induction of autophagy by treatment with trehalose or lithium can decrease pathogenic and infectious PrP^Sc^ expression in persistently prion-infected neurons [25,26]. Moreover, GSS transgenic mice that were treated with rapamycin, an autophagy inducer, exhibited a dose-related delay in disease onset, a reduction in clinical sign severity, and an extension of survival [27]. These results suggest that the administration of an autophagy inducer may be a therapeutically tenable option for treating prion diseases. In addition, an enhanced macroautophagic response was observed in scrapie-infected (strain 263K) hamsters and in human genetic prion diseases [28], indicating that there is a correlation between the up-regulation of autophagy activation and the pathogenesis of prion diseases.

**Figure 1:**
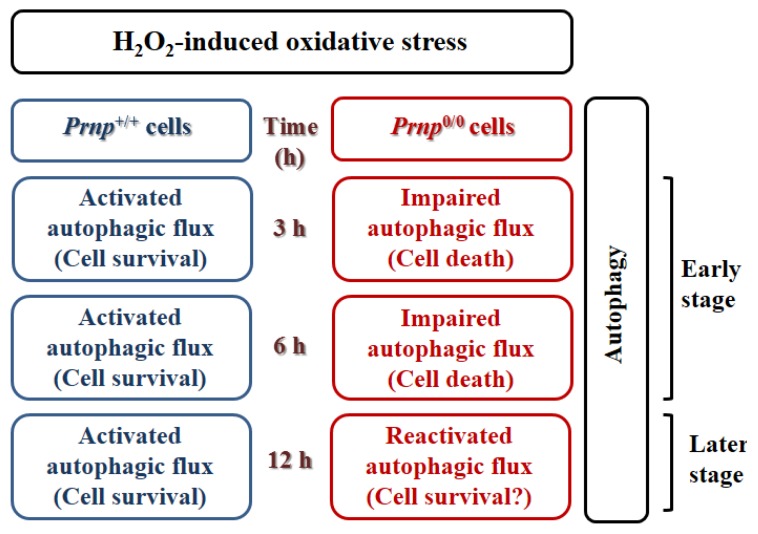
Schematic representation of time-dependent H_2_O_2_-induced cell death in *Prnp*^+/+^ and *Prnp*^0/0^ cells [30].

In addition to autophagy in prion diseases, a correlation between PrP^C^ and autophagy was recently described. Increased expression of LC3-II, autophagy marker protein and autophagosomes were observed in Zürich I *Prnp*^0/0^ hippocampal neurons compared to wild-type control cells under serum starvation [29]. This increased LC3-II was inhibited by the transfection of the wild-type *Prnp* gene into *Prnp*^0/0^ hippocampal neurons, but not by the introduction of PrP^C^ lacking the octapeptide repeat region. Thus, the octapeptide repeat region of PrP^C^ might play a crucial role in the control of autophagy in neurons. Although the autophagic responses of wild-type and *Prnp*^0/0^ hippocampal neurons were clearly different, no definitive association between PrP^C^ and the autophagy pathway was demonstrated in this previous report. In wild-type cells, decreased autophagy induction may be due to the effect that PrP^C^ has on anti-oxidant activity. A recent report revealed a novel protective mechanism that PrP^C^-associated autophagy has against hydrogen peroxide (H_2_O_2_)-induced oxidative stress [30]. Interestingly, autophagy was oppositely regulated in *Prnp*^+/+^ and *Prnp*^0/0^ hippocampal neurons under oxidative stress. In this study, increased autophagy following H_2_O_2_ treatment was due to enhanced and impaired autophagic flux in *Prnp*^+/+^ and *Prnp*^0/0^ hippocampal neurons, respectively. Inhibition of autophagy by siRNA knockdown of *Atg7*, which is essential for the formation of autophagosomes, supports the suggestion that enhanced autophagy led to cell survival in H_2_O_2_-treated *Prnp*^+/+^ cells and that impaired autophagic flux contributed to cell death in H_2_O_2_-treated *Prnp*^0/0^ cells. Moreover, PrP^C^ itself may not be directly involved in autophagic flux in these cells given that a PrP^C^ deficiency did not affect the basal autophagic flux under normal culture conditions without H_2_O_2_ treatment (Figure 1) [30]. 

It was recently reported that the ectopic overexpression of PrP-like protein doppel (PRND) in addition to PrP^C^ deficiency in Ngsk (*NP*^0/0^) mice provokes the impairment of autophagic flux in central nervous system neurons, an effect that is potentially associated with progressive cerebellar Purkinje cell death in these animals [31,32]. PRND alone can cause neurotoxicity, and PRND toxicity is involved in the upregulation of both heme oxygenase 1 (HMOX1) and nitric oxide synthase (nNOS and iNOS) systems, which suggests that there is increased oxidative stress in the brains of the *NP*^0/0^ mice [33,34]. Thus, the defective autophagic flux exhibited by *NP*^0/0^ mice may be due to PRND toxicity-induced oxidative stress.

In human malignant glioma cell lines and non-glial tumor cells, the knockdown of PrP^C^ using antisense oligonucleotides targeting the *Prnp* transcript induces autophagic cell death without the presence of apoptosis markers [35]. This evidence suggests that PrP^C^ may directly modulate the autophagy-dependent cell death pathway.

## 3. The functional role of PrP^C^ on autophagy in vivo

Normal PrP^C^ is highly expressed in the neurons of CNS and especially in their synaptic plasma membrane [3,4,36]. PrP^C^ has several roles in cellular metabolism and maintenance, including neurotransmitter metabolism, signal transduction, copper metabolism, cell adhesion, neuritogenesis, and anti-oxidant activity. Furthermore, many studies have demonstrated that PrP^C^ has neuroprotective and anti-apoptotic functions [37,38,39,40,41,42]. 

Mice in which *Prnp* is ablated have been used in several studies that examined behavior and cognition [43]. More recent studies have noted certain differences between PrP^C^ knockout and wild-type mice. Several important facts have emerged from these studies. For example, PrP knockout mice exhibit increased susceptibility to neuronal damage by oxidative stress and cerebral ischemia. Additionally, neurotoxicity is caused by the expression of Doppel and N-terminally truncated PrP [31,44,45,46]. The multiple effects of PrP deficiency in the same transgenic mouse line suggest its essential function and has broad implications [47].

**Table 1:** A summary of autophagic alteration with aging of Ngsk PrP-deficient mouse line [32].

	3-4 months of age	6-8 months of age
**PrP-deficient mice **	LC3B, P62 and Lamp1 are increased Accumulation of autophagy features in axons and soma and dendrites	LC3B, P62 and Lamp1 are increased Scrg1 accumulates in Golgi apparatus Accumulation of autophagy in the axons, soma and dendrites Increased amount of autophagic degeneration

**Figure 2:**
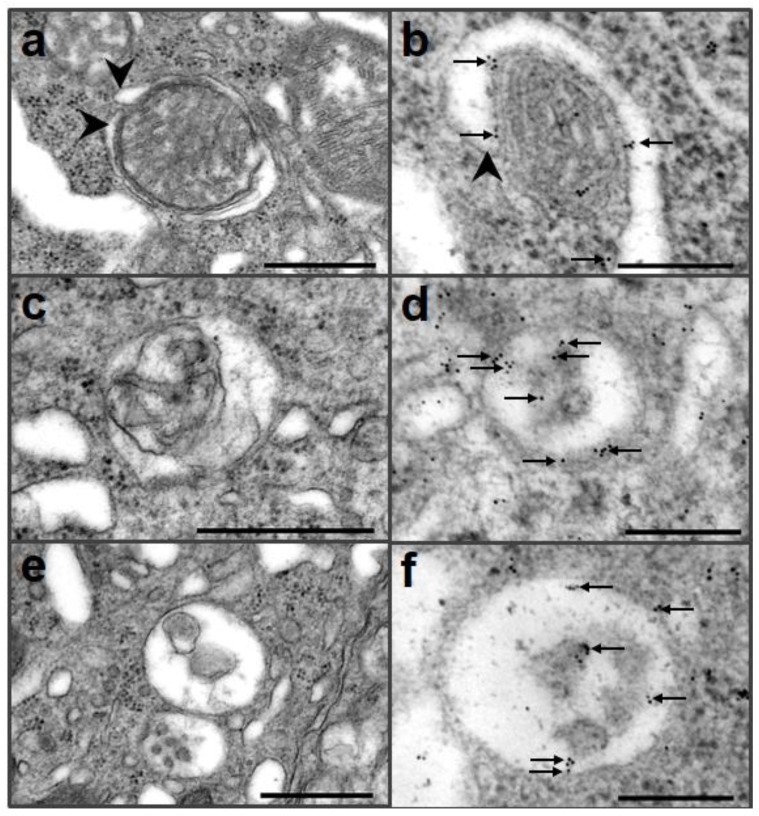
Immunogold electron microscopic analysis of autophagy in Purkinje cells of Ngsk mice using an anti-LC3 antibody. Autophagosomal membranes (arrowheads) began to form vacuoles that contained mitochondria (a, b), and gold particles (arrows) were located in isolated membranes (b). Autophagolysosome showed that single membrane surrounding mitochondrion (c). Immunogold particles (arrows) were located in single membrane and residues (d). Autophagolysosomes were bounded by a single membrane and contained residues (e), and gold particles (arrows) were detected in single membrane and residues (f). Scale bar = 500 nm (a, c, e), 200 nm (b, d, f).

Large deletions in *Prnp*, (e.g., in the Ngsk PrP-deficient mouse line) have neurodegenerative effects in Purkinje cells, which may induce neuronal autophagy [31,32]. Ultrastructural analysis of the Purkinje cells of Ngsk PrP-deficient mouse revealed multivesicular bodies and mitochondria around the autophagic membrane in the soma (Figure 2). Dystrophic axons were also observed that exhibited features of acute autophagy, numerous reticular phagophores, and autophagosomes containing axoplasmic material [32] (Table 1). 

Autophagy is a natural process that removes misfolded proteins, dysfunctional mitochondria, and other potentially toxic proteins or organelles [9]. Although the role of autophagy in neurodegeneration has yet to be established, it is clear that cell death is induced by autophagy in neurodegenerative disorders, such as Alzheimer’s, Huntington’s and Parkinson’s disease (PD), that exhibit protein aggregation [10,48,49]. The results from experiments where autophagy is either inhibited or stimulated indicates that altered protein clearance and organelle clearance, either increased or decreased, are involved in the onset of PD [48]. Furthermore, in the degenerated hippocampus of an early-onset Alzheimer’s mouse model, there are increased protein levels of the autophagy formation marker LC3-II. In addition, actin cytoskeletal and molecular motor defects lead to transport abnormalities and the accumulation of autophagosomes in dystrophic neurites in the hippocampus of these mice [49]. 

The mechanisms of neuronal death have been examined intensively to gain insight into the pathological processes that are associated with acute and chronic neurological illnesses. Prion diseases belong to the family of neurodegenerative disorders that affect both humans and animals. It is known that one of the fundamental steps in the pathogenesis of these diseases is the conversion of the host’s cellular prion protein, PrP^C^, into the disease-associated form, PrP^Sc^ [6,50]. Membrane-anchored PrP^C^ is required to transduce the neurotoxic signals that are elicited by the pathogenic forms of PrP [51]. However, neuronal death is also induced in PrP-knockout mice, and this toxicity is dose-dependently suppressed by the coexpression of full-length PrP [52]. These results suggest that the normal biological activity of PrP^C^ may be altered during the disease process. However, the cellular pathway and molecular components that are involved in this mechanism have yet to be identified.

Various mechanisms have been proposed to explain neuronal death in prion diseases, with apoptosis and autophagy being the most probable types of cell death involved [3,18,22]. Recently, evidence of apoptosis, such as morphologically apoptotic nuclei or cells immunostained with antibody against the activated form of caspase-3, was not detected in prion disease [4]. *In vivo* investigations show contradictory results, especially regarding the function of Bax in neuronal cell death in prion disease [21,26,53]. Dong and coworkers demonstrated that deletion of the proapoptotic protein Bax does not alter either the clinical signs or the Purkinje cell degeneration in Dpl transgenic mice [54]. 

Finally, many studies have reported that a defective autophagy pathway is directly involved in other neurodegenerative disorders, such as Alzheimer’s, Huntington’s, PD, frontotemporal dementia and acute brain injuries [10,48,49,55,56]. However, the evidence for defective autophagy is unclear with respect to prion diseases. Hence, determining the exact role of autophagy in the context of PrP^C^ loss-of-function and prion diseases is likely to contribute to elucidating the pathogenesis of these conditions.

**Figure 3:**
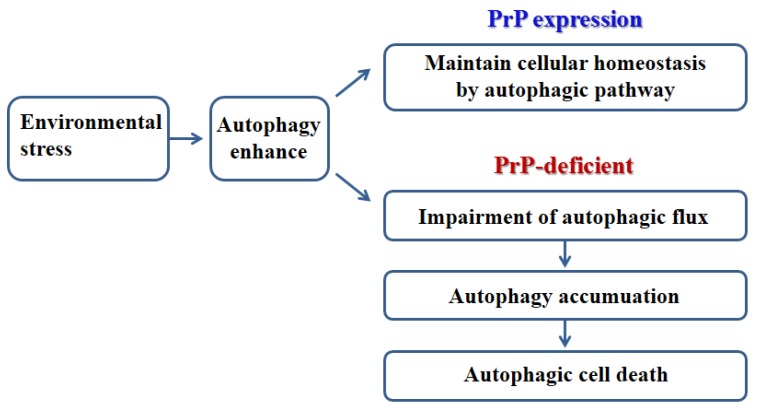
Schematic representation of environmental stress induced autophagic pathway in PrP-expression and PrP-deficient models.

## 4. Conclusions

Decreased autophagy induction may be due to the influence that PrP^C^ has on anti-stress activity in normal cells. The role of PrP^C^-associated autophagy is revealed a novel protective mechanism against oxidative stress [30]. However, in PrP-deficient cells, enhanced autophagy leads to impaired autophagic flux, which contributes to cell death by oxidative stress [30]. The impairment of autophagic flux in CNS neurons is potentially associated with progressive cerebellar Purkinje cell death in the Ngsk PrP-deficient mouse line [30,31,32]. Taken together, the deficiency of PrP^C^ contributes to autophagic neuronal cell death via impaired autophagic flux (Figure 3). 
